# An Inductive Sensing System to Measure In-Socket Residual Limb Displacements for People Using Lower-Limb Prostheses

**DOI:** 10.3390/s18113840

**Published:** 2018-11-09

**Authors:** Katrina M. Henrikson, Ethan J. Weathersby, Brian G. Larsen, John C. Cagle, Jake B. McLean, Joan E. Sanders

**Affiliations:** Department of Bioengineering, University of Washington, 3720 15th Ave NE, Box 355061, Seattle, WA 98195-5061, USA; khenriks@uw.edu (K.M.H.); ethanw3@uw.edu (E.J.W.); bglars@uw.edu (B.G.L.); jcagle23@gmail.com (J.C.C.); jakemc@uw.edu (J.B.M.)

**Keywords:** amputee, prosthetic socket, socket fit, sensor design, residual limb displacements, pistoning, prosthetic socks

## Abstract

The objective of this research was to assess the performance of an embedded sensing system designed to measure the distance between a prosthetic socket wall and residual limb. Low-profile inductive sensors were laminated into prosthetic sockets and flexible ferromagnetic targets were created from elastomeric liners with embedded iron particles for four participants with transtibial amputation. Using insights from sensor performance testing, a novel calibration procedure was developed to quickly and accurately calibrate the multiple embedded sensors. The sensing system was evaluated through laboratory tests in which participants wore sock combinations with three distinct thicknesses and conducted a series of activities including standing, walking, and sitting. When a thicker sock was worn, the limb typically moved further away from the socket and peak-to-peak displacements decreased. However, sensors did not measure equivalent distances or displacements for a given sock combination, which provided information regarding the fit of the socket and how a sock change intervention influenced socket fit. Monitoring of limb–socket displacements may serve as a valuable tool for researchers and clinicians to quantitatively assess socket fit.

## 1. Introduction

Measurements of relative displacements between the residual limb and prosthetic socket may be a valuable clinical tool in monitoring socket fit for people with transtibial amputation. Both daily and long-term changes in residual limb volume and shape alter the coupling between the limb and socket, resulting in degradation of socket fit that manifests as relative motion between the residual limb and prosthetic socket [1,2]. Greater limb–socket displacements are associated with lower perceived socket comfort, greater incidence of skin breakdown, and gait instability.

Prosthesis users commonly adjust their prosthesis to accommodate changes in fit, through actions such as adding or removing socks to reduce socket size or temporarily doffing the prosthesis to allow limb fluid volume recovery. However, such accommodation strategies require the prosthesis user to recognize when changes are needed through indications such as pain, skin breakdown, stumbling, or falls [3]. At-home monitoring of limb–socket displacements may be used to convey to the prosthesis user that an accommodation is needed, and in doing so, prevent the negative consequences from a degraded socket fit. Further, providing practitioners with data on their patients’ socket fit and accommodation strategies may help practitioners better understand and inform their patients, or justify the need for prosthesis modifications or replacement.

A number of sensors have been utilized to investigate socket fit. Load cells mounted beneath the socket provide insight into the forces and moments acting on the socket, but their high power consumption and indirect measurement of limb–socket interactions limit the applicability of the technology for socket fit monitoring applications [4,5,6]. Force sensing resistors (FSRs) have been widely used to measure interface pressures, under the premise that changes in socket fit manifest as increases in pressure magnitude at key weight bearing locations [7,8,9]. However, the locations of focal pressures on a residual limb are localized and highly dynamic, and change based on the socket fit and residual limb fluid volume changes [2]. Further, the poor accuracy and resolution and significant drift exhibited by many FSR models limit the reliability of assessment of actual changes in fit [10,11,12,13].

Researchers have also used commercial displacement sensors to evaluate limb–socket displacements. Photoelectric [14] and inductive [15] sensors were placed at the inferior aspects of prosthetic sockets to evaluate vertical limb motion (i.e., pistoning) during ambulation. While the results of these studies provided meaningful insight into pistoning magnitudes, the sensors used were bulky, unsuitable for long-term monitoring, and required permanent modification of the participants’ sockets.

A smaller, battery-powered inductive sensor has enabled longer-term monitoring outside of the laboratory [16,17]. Using a target made of conductive fabric that was adhered to elastomeric liners, the sensor measured limb–socket displacements during out of lab tests ranging from two days to two weeks. However, debris build-up between fibers of the target material caused significant signal degradation [16,17]. To address the degradation challenges associated with conductive fabric targets, a novel iron-seeded polymeric target was developed and incorporated into prosthetic sheaths worn over elastomeric liners [18]. The ferromagnetic composite sheaths performed better than conductive targets, reducing signal degradation from 50% in two weeks to less than 3% over four weeks [16,18]. However, the sheath was susceptible to bunching and it added thickness to the liner that some participants found uncomfortable.

Previously, the inductive sensors were incorporated into individual polymeric shells or 3D printed inserts that were temporarily adhered to the inner socket wall. Individual polymeric shells allowed for quick instrumentation, but may have altered the interface mechanics between the limb and socket. The fabrication of 3D printed inserts was a time-consuming process and participants needed to have a loose-fitting socket, so that the inserts took up space normally occupied by socks. Otherwise, a larger socket had to be fabricated to fit the inserts [17].

This research expands on prior investigations to develop and evaluate a novel, wearable inductive sensing system to measure the distance between the residual limb and the prosthetic socket. Sensors were embedded into cable-paneled adjustable sockets for participants with transtibial amputation, and the ferrous target technology was integrated into elastomeric prosthetic liners. Benchtop and clinical tests were performed to assess the integrated sensing system’s ability to measure limb–socket distances and displacements.

## 2. Materials and Methods

**Figure 1:**
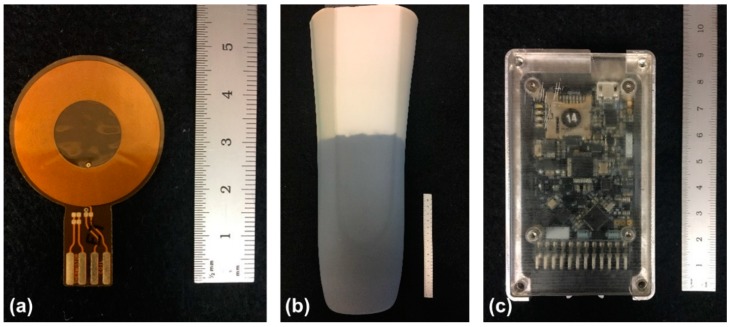
Inductive sensing system components: (**a**) sensor antenna; (**b**) inner surface of ferrous liner with iron-doped polymer between fabric and normal (unfilled) elastomer; (**c**) portable data acquisition unit.

The wearable sensing system consisted of a low-profile sensor, a flexible iron-seeded elastomeric target, and a portable data acquisition unit (Figure 1) [16,17,18]. Sensors were embedded into prosthetic sockets and flexible targets were created by incorporating iron particles into elastomeric prosthetic liners so that the sensors measured the distance between the liner and socket wall.

The low-profile sensor was a custom-designed flexible coil antenna (diameter 32.0 mm, thickness 0.15 mm) and a surface-mounted capacitor (220 pF). A 10 kΩ surface mount thermistor was soldered to the antenna to monitor the temperature of the sensing environment and compensate for thermal-induced drift.

A custom-designed portable data acquisition unit containing an inductive sensing chip (LDC1614, Texas Instruments, Dallas, TX, USA) was used to power the sensors and collect proximity data. When powered, the inductor and capacitor operated as an inductor–capacitor (LC) tank oscillator. The presence of the magnetically permeable target within the sensor’s field reinforced the inductor and lowered the sensor’s oscillation frequency in a distance-dependent manner. Therefore, the changes in sensor frequency measured by the inductive sensing chip were a sensitive measure of proximity between the target and sensor antenna. The sensor output (proximity counts) is a ratio of the sensor’s oscillation frequency to an external reference clock frequency.

The wearable sensor target was a ferrous elastomeric liner worn over the residual limb. The liner was constructed so that the iron-doped polymer (thickness 1 mm, iron content 80 percent by weight) was embedded between the liner fabric and the normal (unfilled) elastomer.

**Figure 2:**
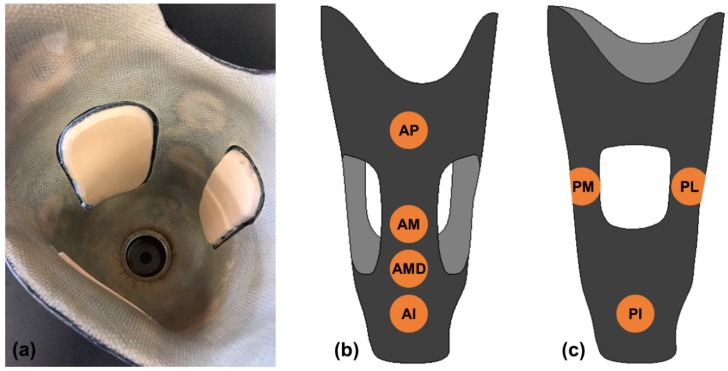
Sensors embedded into seven locations in the prosthetic socket: (**a**) socket with embedded sensors visible through Nyglass inner layup; (**b**) sensor locations of the anterior aspect of the socket; (**c**) sensor locations of posterior socket. Sensors anterior midlimb (AM), posterior midlimb medial (PM), and posterior midlimb lateral (PL) were fabricated on a latitudinal line at half the average height of each panel. Anterior proximal (AP); anterior midlimb distal (AMD); anterior inferior (AI); posterior inferior (PI).

Instrumented, adjustable cable-paneled sockets were fabricated for participants with transtibial amputation. Each participant’s regularly-used socket was digitized using a mechanical coordinate measurement machine (FaroArm Platinum, FARO Technologies, Lake Mary, FL, USA) so that the instrumented socket duplicated the shape of the current socket. Sensors were embedded between an inner layup consisting of four layers of Nyglass stockinet (Paceline, Matthews, NC, USA) and epoxy acrylic resin (Paceline, Matthews, NC, USA) and a secondary layup consisting of a single layer of carbon fiber. This was followed by a final four-layer outer layup consisting of two layers of carbon fiber separated by two layers of Nyglass. Tubing and cabling for the panels was placed between the secondary and outer layups. Ferrite shielding (thickness 0.3 mm, Wurth Electronics, Waldenburg, Germany) was attached to the outer-facing side of the inductive sensors to block electromagnetic interference from the carbon fiber and external environment. Sensors were placed in the anterior proximal (AP), anterior midlimb (AM), anterior midlimb distal (AMD), anterior inferior (AI), posterior inferior (PI), posterior midlimb medial (PM), and posterior midlimb lateral (PL) aspects of the socket (Figure 2). The anterior midlimb distal sensor was omitted for participants with short residual limbs. A cable connected the panels of the socket such that by extending or retracting the cable, the panel distances relative to the socket could be adjusted.

Calibration of the embedded sensors was conducted in two stages; a detailed benchtop calibration followed by a reduced-point in-socket calibration. Preliminary sensor repeatability tests revealed that the sensor configuration (i.e., depth in layup, sensor curvature) shifted the sensor sensitivity curve; therefore, each sensor should be calibrated in its final embedded in-socket configuration (Appendix A). Variation in the thickness of the embedded iron layer in the ferrous liners caused a similar shift in sensor response, indicating that the in-socket calibration needed to be performed with the ferrous liner matching the socket of interest (Appendix A). The dual-stage calibration procedure was conducted to determine the offset between a single, detailed calibration and the unique in-socket response due to variation in sensor configuration and target variability. This dual-stage calibration also minimizes any error associated with inconsistency in the target liner during in-plane movements of the limb, which were not assessed in this study but have been addressed previously [18].

**Figure 3:**
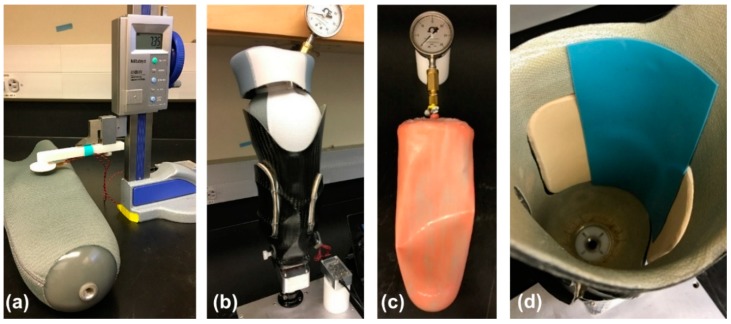
Calibration systems: (**a**) benchtop calibration system consisted of a sensor fastened to the arm of a vertical height gauge. A high-resolution measurement was obtained from a single location on the liner. (**b**) Complete in-socket calibration system with the bladder and liner expanded to force the liner to conform to socket wall while data were collected. (**c**) Bladder used to expand the liner. (**d**) Flexible offset pieces were adhered to the socket wall to measure sensor response at multiple liner–antenna distances.

In the first phase of the calibration procedure, a detailed calibration was obtained for a single location on a ferrous liner using a benchtop setup (Figure 3). The liner was placed flat on the bench with a Delrin^®^ block separating the two sides of the liner and isolating the region of interest. A sensor was fastened to the arm of a digital height gauge (Mitutoyo 570-312, Aurora, IL, USA) so that the height gauge measured the distance between the sensor and the target. Data were collected while the sensor was raised 20 mm away from the liner in steps of 0.25 mm from 0 to 5 mm, 0.5 mm from 5 to 15 mm, and 1 mm from 15 to 20 mm. The sensor was then lowered back into contact with the liner with the same step pattern. The benchtop calibration took approximately 10 min to complete.

An in-socket calibration procedure was then performed to measure the sensitivity of the embedded sensors in their final configuration. In-socket sensor response was assessed at four known distances (0.00 mm, 1.09 mm, 2.19 mm, and 3.29 mm). A custom silicone bladder with a proximal tubing port was placed inside the ferrous liner and was inflated to 27.6 kPa to expand the liner to conform to the socket’s contours (Figure 3). Polymeric offset pieces were fabricated from a Shore 60A platinum cure silicone (PlatSil 73-60, Polytek Development Corp., Easton, PA, USA) to restrict liner expansion and obtain measurements at non-zero distances. Measurements were taken by placing the desired number of offset pieces on the inner socket wall and then inflating the bladder and liner to conform to the socket contours. The in-socket calibration procedure took approximately 5 min to complete for a single socket.

In-socket calibration data were offset along the x-axis from the benchtop calibration data. Offsets were calculated by converting the proximity counts measured during the in-socket calibration into distances using the unadjusted benchtop calibration data. Calculated distances were subtracted from the actual socket–liner distances (thickness of the offset pieces), and the median of these differences was taken to obtain a single distance offset for each sensor. Offsets were applied to the benchtop calibration to create individual calibrations for each embedded sensor location that reflected the sensor’s in-socket response.

Participants were included in this study if they had a transtibial amputation at least 18 months prior and regularly used a definitive prosthesis at least four hours per day without assistive aides. Candidate participants were excluded if they presented with skin breakdown or soft tissue injury at the time of study. Study procedures were conducted in accordance with approval #49624 from the University of Washington Institutional Review Board. All participants provided written informed consent prior to any study procedures being performed.

Participants conducted two in-lab procedures to assess the ability of the sensors to measure limb–socket distances and displacements and evaluate socket fit. In the first portion of the test session, participants were asked to don a variety of sock combinations and stand with equal weight bearing for 15 s for each combination. Thickness of each sock combination under incremental loading up to 101.2 kPa was tested after the session using a tabletop test system [19,20]. The researcher then asked participants to identify a minimum and maximum sock thickness in which they could safely walk, and selected an intermediate sock thickness between the self-selected minimum and maximum. In the second portion of the test session, participants conducted a series of activities while wearing each sock combination. The activities were as follows: stand (15 s), walk (1 min), stand (15 s), sit (1 min), stand (15 s), walk (1 min), stand (15 s), and sit and change sock.

The distance between the limb and socket during the first portion of the test was obtained for each of the three sock combinations as the average of the distance measurements over the 15-s standing period. Peak-to-peak displacements were calculated for the walking portion of the test session as the difference between the maximum and minimum distance for a step. The minimum distance during a walking cycle represented the limb–socket distance during the stance phase of gait, whereas the maximum distance represented swing phase. Displacements were calculated for each step, and averaged over each walking cycle to obtain one average peak-to-peak displacement per sock combination.

## 3. Results

### 3.1. Sensor Calibration

Calibration of a single location on a ferrous liner with the benchtop calibration system revealed that the sensor effectively measured the distance between the sensor and target over a 15 mm range, with a full-scale output (FSO) of 7.6 × 10^5^ counts. No substantial hysteresis was observed. The sensor was more sensitive at closer distances, with sensitivity ranging from 2.2 × 10^5^ counts/mm at the closest distances to 9.2 × 10^2^ counts/mm at the farthest distances. The peak-to-peak noise during the calibration steps averaged 0.06% FSO, and the drift measured over a 30 min period was 0.08% FSO. A fifth-order polynomial fit well to the data, with the root mean square (RMS) error within 0.007% FSO.

**Figure 4:**
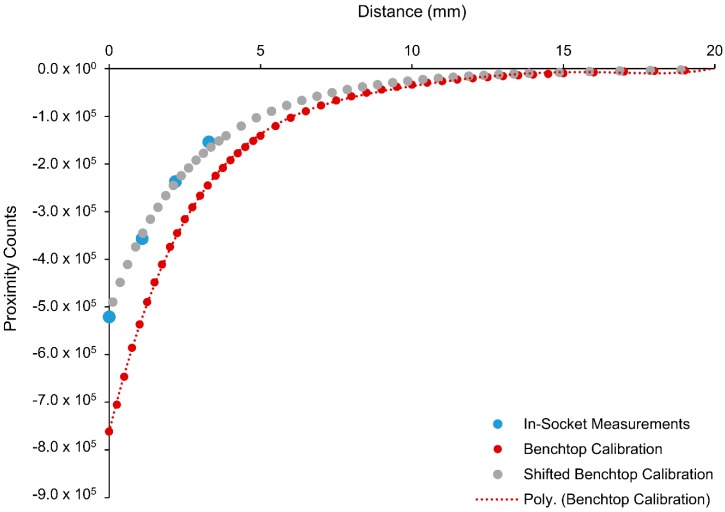
Benchtop calibration obtained from one location on a ferrous liner, in-socket measurements from one sensor in the socket for Participant 1, and the x-axis offset-corrected benchtop calibration to accurately represent the embedded sensor’s response.

In-socket calibrations revealed that embedded sensors had calibration curves that were consistent in shape with each other and the benchtop calibration, but were shifted along the x-axis (distance) (Figure 4). The x-axis offsets between the embedded sensor calibrations and the benchtop calibration ranged between −0.4 mm and 3.1 mm. When the x-axis offset correction was applied to the benchtop calibration, the RMS error between in-socket measurements and the calibration curve averaged 2.78% FSO across all sensors in four test sockets (*n* = 27 sensors). Conversely, the RMS error between the unadjusted benchtop calibration curve and the in-socket measurements averaged 26.83% FSO, highlighting the importance of calibrating each embedded sensor to minimize calibration errors.

### 3.2. Clinical Evaluation

Four people with transtibial amputation of traumatic etiology participated in this study. Three of the participants were male and one participant was female. The mean age of the participant pool was 48 ± 22 years, and the mean time from amputation was 21 ± 16 years. All participants were K-3 level ambulators and used total surface bearing prostheses with locking pin suspension. Participant 2 and Participant 4 had hypertension, a risk factor for vascular disorders that influence residual limb fluid volume [1,21]. The panels of the socket were in a neutral position, that is, the inner face of the panels was flush with the surrounding socket wall, for three of the four participants. Participant 4 could not wear socks with the panels in the neutral position, so 16.63 mm of cable was added to the socket to loosen the panels and increase the socket size by 2.44%. The panels remained in a single position for the entirety of the testing session.

**Figure 5:**
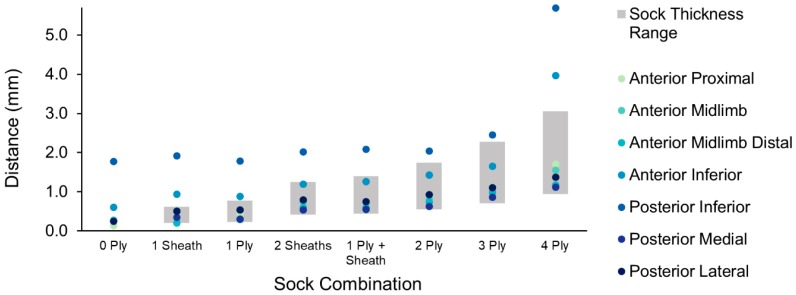
Sensor measured limb–socket distances and actual sock thickness range for each sock combination worn by Participant 1. The sock thickness range was measured between 0 and 121.5 kPa using a benchtop measurement system. Sensors did not measure equivalent distances for a single sock combination. Distances greater than the maximum sock thickness indicated an air gap between the limb and liner, whereas closer distances indicated greater compression of the sock over the sensor.

Limb–socket distances measured during standing with equal weight bearing were compared with the corresponding thickness range of the sock combination. Sensor-measured distances increased with sock thickness, but sensors did not necessarily measure equivalent distances for a given sock combination (Figure 5). Distances greater than the maximum unloaded sock thickness were attributed to the presence of an air gap between the sensor and liner, whereas closer sensor-measured distances indicated greater compression of the sock. For all participants, the posterior inferior sensor commonly measured distances greater than the maximum sock thickness, indicating an air gap between the socket and liner at the distal end of the socket. Each participant had one sensor that consistently measured the closest distances, but this sensor varied by participant (posterior medial for Participant 1, anterior midlimb for Participants 2 and 3, and anterior inferior for Participant 4).

**Figure 6:**
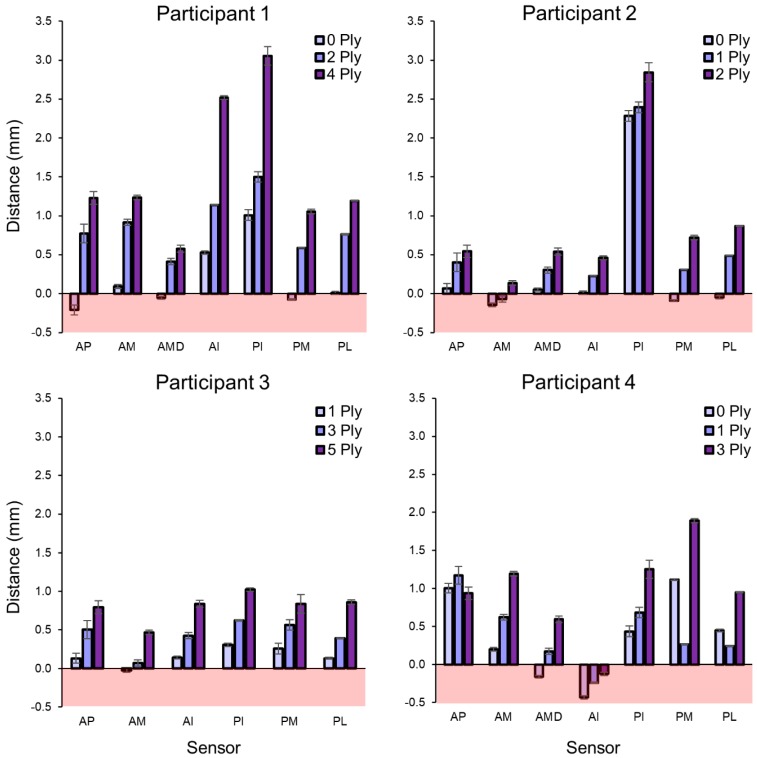
Average walking stance phase distances between the limb and socket by sock combination. As sock thickness increased, the limb typically moved further away from the socket wall. Distance measurements less than 0.0 mm indicate that the liner was under compression.

When participants wore thick socks compared with thin or no socks while walking, stance phase limb–socket distances increased, barring discrepancies at three sensors for Participant 4 (Figure 6). For that participant, the limb moved away from the socket wall in the anterior proximal region and closer in the posterior medial and lateral regions when a 1 ply sock was added. Such a change indicates that the addition of the sock significantly altered the fit of the socket, shifting loading from the anterior aspect of the socket to the posterior midlimb region. The substantial increase in stance phase distance for the inferior sensors for the 4 ply condition for Participant 1 suggests that the addition of the thicker sock caused the limb to sit higher in the socket and was likely “hung up” on the proximal aspects of the socket. Negative stance phase distances were likely the result of compression of the ferrous liner against the socket wall, as demonstrated in Appendix A.

**Figure 7:**
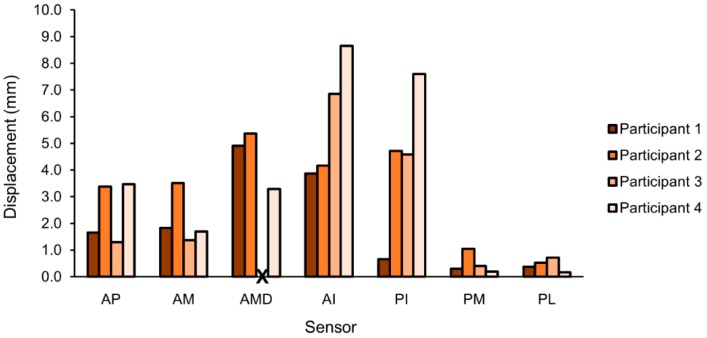
Peak-to-peak limb–socket displacements when participants wore an intermediate sock thickness. Participants had unique displacement profiles, with varied displacement magnitudes by sensor. Participant 3 did not have an anterior midlimb distal sensor.

Peak-to-peak limb–socket displacements, that is, the difference between the swing phase and stance phase distances, were compared across participants and sock combinations during the walking trials. The displacement profile was unique for each participant (Figure 7). The posterior medial and lateral midlimb sensors consistently measured the smallest peak-to-peak displacements. The greatest peak-to-peak displacements were measured at the anterior midlimb distal sensor for Participant 1 and Participant 2 and at both inferior sensors for Participant 3 and Participant 4.

**Figure 8:**
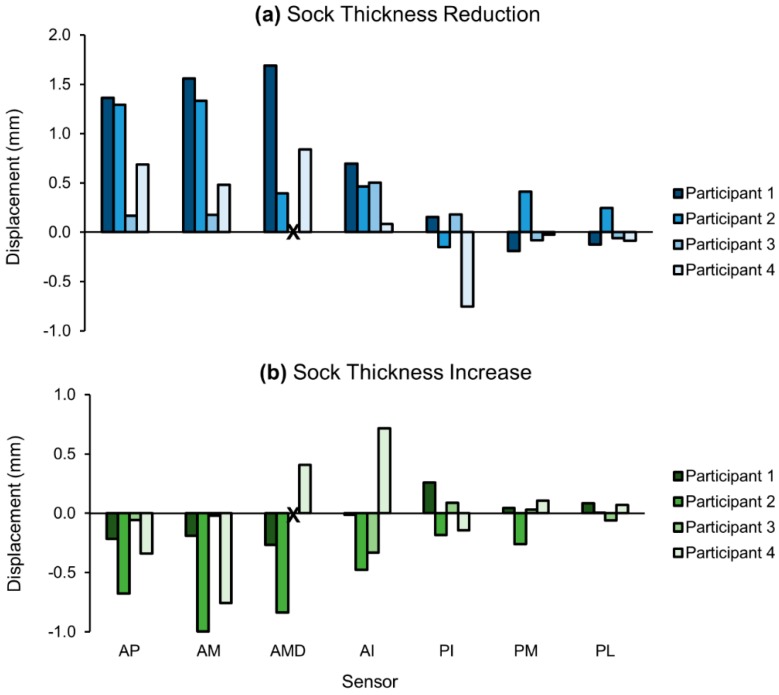
Changes in peak-to-peak limb–socket displacements for the following: (**a**) a reduction in sock thickness from intermediate; (**b**) an increase in sock thickness from intermediate. Displacements typically increased when sock thickness decreased and decreased when sock thickness increased. Participant 3 did not have an anterior midlimb distal sensor.

Peak-to-peak displacements typically decreased as sock thickness increased; however, the changes following a sock change were relatively small (Figure 8). When participants wore a thinner sock than the intermediate condition, the minimum and maximum changes in peak-to-peak displacements were −0.8 mm and 1.7 mm, respectively. The minimum and maximum changes when a thicker sock was worn were −1.0 mm and 0.7 mm, respectively. The mean displacement change magnitude was 0.42 mm for a sock thickness reduction, and −0.2 mm for a sock thickness increase. Not all sensors measured changes in displacement that followed the trend of lower displacements with thicker socks and greater displacements with thinner socks, indicating a non-global change in fit with the sock change.

## 4. Discussion

The purpose of this study was to evaluate an embedded sensing system that measured the distance between the socket and the residual limb. This sensing system may serve as a useful tool for researchers to investigate how interventions or new technologies influence socket fit. For clinicians, monitoring of residual limb displacements in the clinic or longer-term data collection in a patient’s free-living environment may help diagnose socket fit problems, and justify a new socket as needed.

Laminating sensors into the socket wall and embedding the target material into prosthetic liners overcame limitations of prior designs, allowing many sensors to be used without disturbing the normal limb–socket interface [16,17,18]. The quick in-socket calibration procedure that accounted for each sensor’s unique position and curvature within the socket further contributed to the practicality of the design by reducing the error to less than 3% FSO. Measured peak-to-peak displacements at the anterior and inferior sensors ranged from 0.7 mm to 9.4 mm, respectively, consistent with findings from prior studies [14,15,17,22].

The enhanced capabilities of the system provide new clinical insight. The results demonstrated that prosthesis users and practitioners should not expect the addition of a sock to alter the distance between the limb and socket in a uniform manner during standing and walking. The sock may cause the relative limb position to change, resulting in localized contact between the limb and socket in some regions or loss of contact in other regions. Participant 1 in the present study appeared to shift upward in the socket when the maximum sock thickness was added, evidenced by substantially greater limb–socket distances at the anterior inferior and posterior inferior sites. Participant 4, on the other hand, may have experienced a posterior translation of the limb when the intermediate sock thickness was added, resulting in closer distances measured by the posterior midlimb sensors. Similar to interface stress measurements reported in the literature, the displacement distribution and changes in this distribution with an intervention varied among participants [23,24,25,26]. This finding further highlights the complexity of quantifying degradation of socket fit.

The relatively small changes in peak-to-peak displacements, that is, −0.8 to 1.7 mm, for different sock thickness is surprising. Experienced clinicians can visually identify “pistoning” during gait inspection when socket fit has degraded, which would indicate that larger displacements occur. However, pistoning is movement of the limb relative to the socket and may primarily reflect displacement between the bone and surrounding tissue, as opposed to displacement between the limb and socket. A number of researchers have utilized imaging techniques such as radiography and motion analysis to investigate relative bone–socket positions in both static and dynamic positions [27]. Across these studies, liner–socket displacements (0–16 mm) were typically much smaller than the bone–socket displacements (3–81 mm) [27]. In 1977, Burgess was surprised to find that patients tolerated 19 mm of bone–socket pistoning without disturbed patient comfort or gait [28]. The magnitude of displacement between the liner or skin and the socket that is clinically relevant may thus be smaller than bone–socket displacements typically used to evaluate pistoning.

It will likely be through studies conducted on many prosthesis users that utilize clinical knowledge towards data interpretation that limb–socket measurement technology will advance as a useful diagnostic and prognostic tool for prosthetic care. Technical developments such as incorporating the ferrous polymer targets into any liner model will facilitate such large-scale studies. Participant 1 and Participant 2 normally wore a liner from another manufacturer, and so the fit between the liner and socket was altered using this only-available models of ferrous liner.

The ferrous liners demonstrated susceptibility to compression, resulting in negative distance measurement in some participant test data (Figure 6). It may be possible to control stiffness of the polymer on the outside of the liner between the ferrous target and socket wall, creating the elastic element of a pressure sensor. This advancement would allow the sensor to measure limb–socket distances when the limb is just in contact or further away from the socket and limb–socket pressures when it is forced against the socket wall.

## 5. Conclusions

This study demonstrated the performance of a sensing system designed to measure residual limb distances at multiple locations in the prosthetic socket without disturbing the limb–socket interface. Using a novel calibration method, many sensors were calibrated quickly, contributing to a practical design. The sensors provided insight into limb-socket positions during stance phase and displacements (pistoning) during swing phase. This sensing system may serve as a valuable tool for both researchers and clinicians to understand socket fit as it relates to accommodation strategies and interventions, and patient prosthesis use.

## Figures and Tables

**Figure A1:**
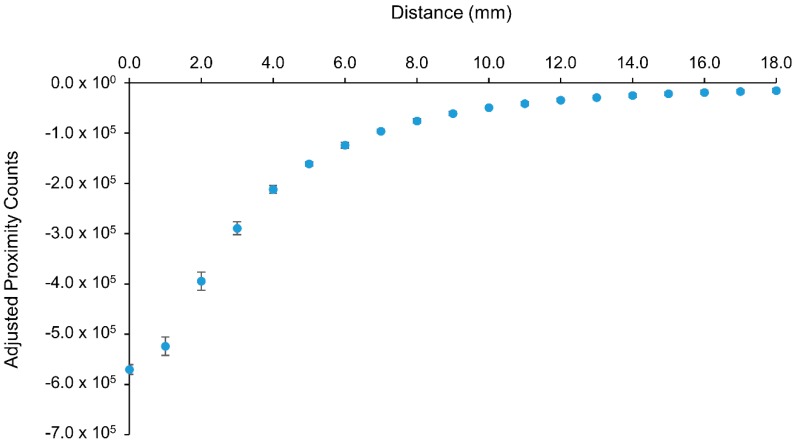
Sensor sensitivity was measured for multiple sensors to assess repeatability. Average sensitivity for the same three sensors after the sensor proximity counts were normalized to set the baseline counts to zero. Curves were shifted along the y-axis when the baseline offset was removed, which minimized repeatability error.

**Figure A2:**
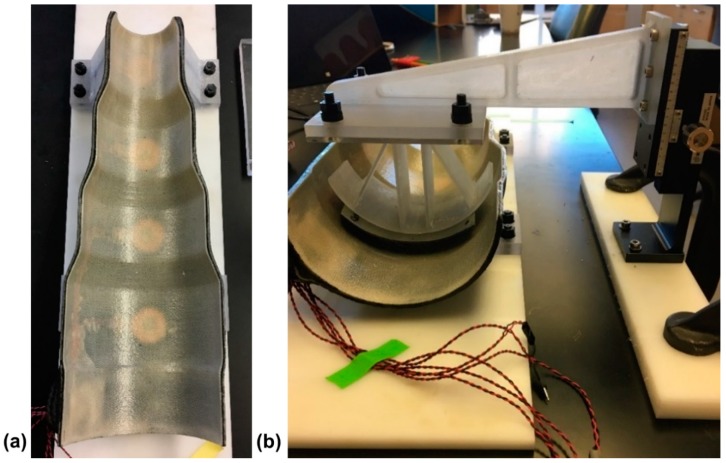
Benchtop setup to evaluate effects of curvature on sensor output: (**a**) composite with embedded sensors; (**b**) 3D printed mounding pieces, probe arm, and z-axis stage used to hold the target in a defined curvature at specified distances from the base.

**Figure A3:**
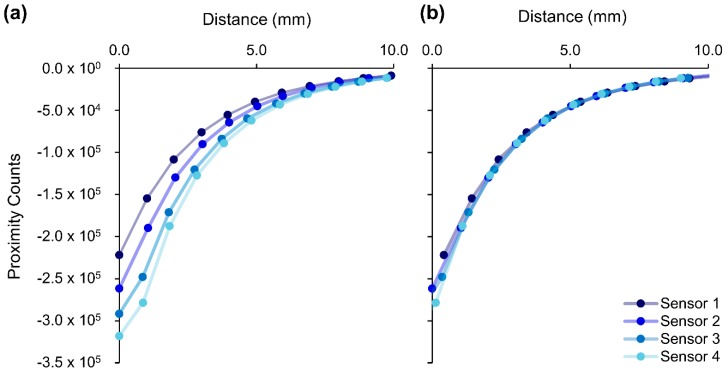
Sensitivity of sensors with varying radius of curvature: (**a**) unadjusted sensor output; (**b**) shifted sensitivity curves aligned well when an x-axis offset was applied.

**Figure A4:**
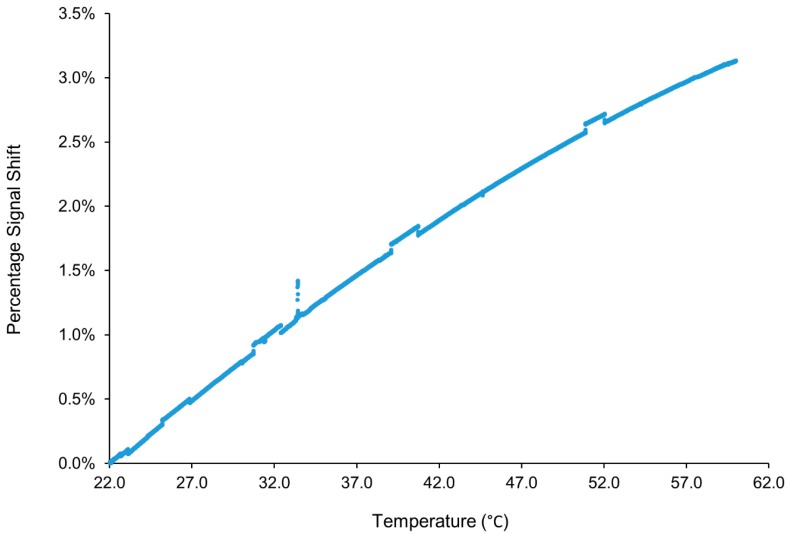
Sensor thermal sensitivity. Sensor oscillation frequency, and thus proximity counts, were linearly proportional to the temperature of the sensing environment.

**Figure A5:**
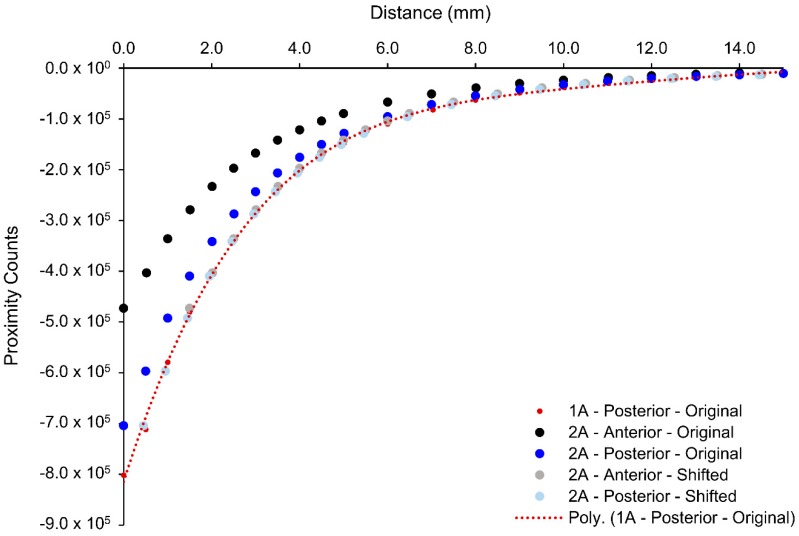
Liner repeatability evaluations. Sensitivity curves aligned well after an x-axis offset was applied.

**Figure A6:**
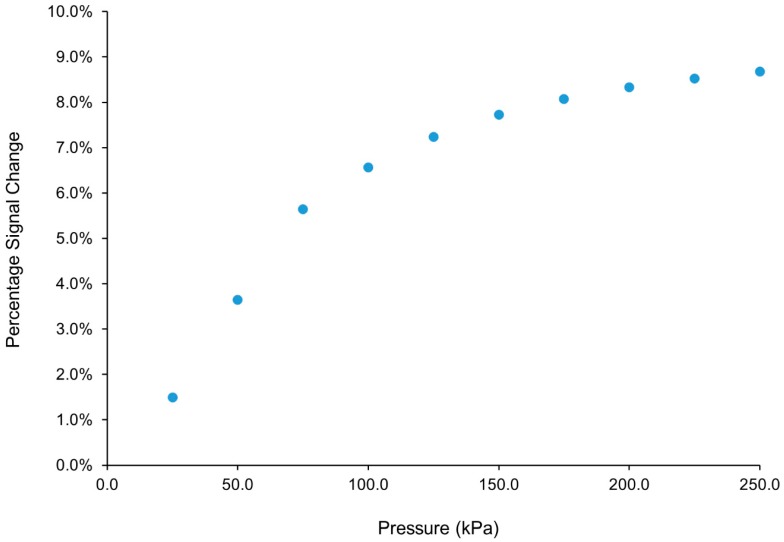
Compression of the ferrous liner at a fixed distance increased the signal.

## Supplementary Materials

The following are available online at http://www.mdpi.com/1424-8220/18/11/3840/s1.

## Appendix A

### Appendix A.1. Antenna Repeatability

Three antennae were randomly selected for evaluation. Sensor sensitivity was measured by adhering the antenna to the arm of a vertical height gauge, which measured the distance between the sensor and a target that was flat on the bench. A single patch of the ferrous target was used in the sensitivity tests to eliminate error associated with consistency between target samples. Sensors demonstrated unique baseline proximity counts when no target was in the sensors’ field of view, indicating that each sensor operated at a different tank oscillation frequency when powered. This resulted in a large standard deviation when the average sensitivity was plotted (Figure A1). This variation was accounted for by subtracting the baseline offset from sensor data, so that the sensor baseline was zero. Removal of the baseline offsets aligned the sensitivity curves by shifting the curves along the y-axis, and reduced error between sensor response curves to an RMS error less than 2.0% FSO.

### Appendix A.2. In-Socket Sensor Configuration

A benchtop test setup was created to investigate the influence of sensor configuration variables on sensitivity (Figure A2). The setup was created with the same inner and outer layup procedure utilized to create the instrumented sockets, and contained four regions with specific radii (3.0 cm, 4.0 cm, 5.0 cm, and 6.0 cm) that were representative of the magnitude of curvature present in prosthetic sockets. One sensor was embedded in each region. These regions were labeled Sensor 1 (3.0 cm), Sensor 2 (4.0 cm), Sensor 3 (5.0 cm), and Sensor 4 (6.0 cm). A single patch of a ferrous target was used to evaluate sensor response. Attachments matching the curvature of each region and fastened to a probe arm and z-axis stage held the target patch in a conforming curvature. The z-axis stage (Misumi ZWG90, Schaumburg, IL, USA) moved the target, and a vertical height gauge (Mitutoyo 570-212, Aurora, IL, USA) with a precision of 0.01 mm measured the position of the z-axis stage and thus the distance between the target and the layup wall. Sensor response was evaluated from 0.0 mm to 10.0 mm in steps of 1.0 mm. The target was then lowered from 10.0 mm to 0.0 mm in 1.0 mm steps to assess hysteresis.

The results indicated that as curvature of the sensor increased, the sensor response curve shifted along the x-axis (distance) (Figure A3). X-axis offsets were applied to align the curves for Sensor 1, Sensor 3, and Sensor 4 with Sensor 2. The offset magnitudes were 0.43 mm for Sensor 1, −0.49 mm for Sensor 3, and −0.73 mm for Sensor 4. After alignment, the RMS error between the curve for Sensor 2 and the shifted curves averaged 1.35% FSO.

### Appendix A.3. Antenna Thermal Sensitivity

The thermal sensitivity of the antenna was evaluated to identify the magnitude and linearity of thermal drift. An antenna was placed in an oven and the temperature was increased from 22 °C to 60 °C while data was collected from the antenna and a surface-mounted thermistor on the antenna. Sensor output was dependent on temperature, and changed by 3.13% across the 38 °C range (Figure A4).

### Appendix A.4. Liner Repeatability

Both within-liner and between-liner repeatability were evaluated by calibrating an anterior midlimb location and a posterior midlimb location on five liners using the vertical height gauge benchtop calibration method. Sensor response was evaluated at antenna-target distances of 0.0 mm to 15.0 mm. The results demonstrated variability in sensor response both between locations on a single liner and between liners that manifested as an x-axis shift of the sensor response curve, similar to the effect of sensor configuration on the response curve (Figure A3). After adjusting the sensor response curves with x-axis offset corrections to align with the curve of one location (Figure A5), the RMS error averaged 0.96% FSO. These findings indicated that the variation in target signal strength needed to be accounted for in the calibration procedure, but that a single calibration curve could be used as a base calibration, similar to the shifting procedure to account for sensor configuration variation.

### Appendix A.5. Liner Compression

The influence of compression of the deformable target-embedded liner on sensor response was assessed using a previously described custom fixture [18]. Compressive stresses up to 250 kPa were applied to a 7.7 cm by 7.7 cm sample of a ferrous liner at an antenna–liner distance of 1.5 mm. The antenna–liner distance of 1.5 mm was selected as it most closely represented contact between the liner and socket wall, with the inner Nyglass layup separating the liner and sensor during contact. Additionally, this distance prevented deformation of the sensor itself during the tests. The signal amplitude was sensitive to changes in liner compression. An increase in the compressive stress to 250 kPa increased the signal amplitude from the uncompressed state by 8.7% (Figure A6). The change in counts at a fixed distance because of compression indicates that when participant session data are calibrated from counts to millimeters, negative distance measurements may occur. With further characterization, this response to compression may allow proximity counts to be converted to pressure; however, because participants wore socks over the liner during the test sessions, more investigation is required to characterize the sensor response to compression of socks in conjunction with the liner.
